# A study of Genomic Prediction across Generations of Two Korean Pig Populations

**DOI:** 10.3390/ani9090672

**Published:** 2019-09-11

**Authors:** Beatriz Castro Dias Cuyabano, Hanna Wackel, Donghyun Shin, Cedric Gondro

**Affiliations:** 1 Department of Animal Science, Michigan State University, East Lansing, MI 48824, USA; 2 The Animal Molecular Genetics and Breeding Center, Chonbuk National University, Jeonju 54932, Korea

**Keywords:** animal breeding, SNP-chips, genomic evaluation, generations

## Abstract

**Simple Summary:**

Commercial genotyping has become accessible at a relatively low cost and nowadays it is widely used by breeders to predict production and economic traits. Many studies explored the benefits of using DNA information in breeding programs, and many methods have been established to optimize the use of such information. To date, however, very few studies have explored how prediction accuracies change across generations. Here we present a short evaluation across five generations in two pig breeds and evaluate the accuracy of the prediction of relevant production traits using different generational groups.

**Abstract:**

Genomic models that incorporate dense marker information have been widely used for predicting genomic breeding values since they were first introduced, and it is known that the relationship between individuals in the reference population and selection candidates affects the prediction accuracy. When genomic evaluation is performed over generations of the same population, prediction accuracy is expected to decay if the reference population is not updated. Therefore, the reference population must be updated in each generation, but little is known about the optimal way to do it. This study presents an empirical assessment of the prediction accuracy of genomic breeding values of production traits, across five generations in two Korean pig breeds. We verified the decay in prediction accuracy over time when the reference population was not updated. Additionally we compared the prediction accuracy using only the previous generation as the reference population, as opposed to using all previous generations as the reference population. Overall, the results suggested that, although there is a clear need to continuously update the reference population, it may not be necessary to keep all ancestral genotypes. Finally, comprehending how the accuracy of genomic prediction evolves over generations within a population adds relevant information to improve the performance of genomic selection.

## 1. Introduction

Genomic models that incorporate dense single nucleotide polymorphism (SNP) marker information are widely used for the prediction of genomic values [1] to select animals and plants in breeding programs [2,3,4,5] or to predict susceptibility to diseases in humans [6,7].

Within a genotyped population, the extent of the genomic relationships [8,9], linkage disequilibrium (LD), and co-segregation of QTL with markers [10] contribute to the accuracy of genomic predictions. In livestock populations such as beef and dairy cattle, sheep, and pigs, the level of relatedness between individuals is higher compared to the relatedness observed in other populations, such as humans.

Genomic relationships are higher within families, rather than across families, and genomic prediction in breeding populations generally relies on the genotypes and phenotypes from ancestral individuals to predict the breeding values of subsequent generations. In fact, this practice of using information from previous generations to predict the next one has been in use for many years and predates the genomic technologies. Before genotypes were available, only the known family relationships from the current and previous generations (in the form of a pedigree relationship matrix) were combined with recorded phenotypes, to predict breeding values.

When genomic prediction is performed over successive generations of the same population, the prediction accuracy is expected to decay if the reference population is not updated, even if the base population initially genotyped was large, due to the decline in the extent of the relationships between reference and test individuals, and the breakdown of the linkage disequilibrium (LD) between SNPs and the quantitative trait loci (QTL) across generations [11].

The practical challenges of updating the reference population across generations to find a balance between genotyping costs and the economic gain from a more accurate selection was discussed by Pszczola and Calus [11], where it was empirically evaluated how many individuals should be updated in the reference population in each generation, in order to maintain a stable prediction accuracy. That evaluation was performed on a simulated population that resembled the genetic structure of dairy cattle. We are not aware of studies reporting results with real data across a large number of genotyped individuals for all generations. Weng et al. [12] evaluated a large number of generations in a layer chicken population, however most of the predictions were pedigree-based, rather than genotype-based.

In this study we performed an empirical evaluation of the prediction accuracy of genomic breeding values for two small populations of Korean pigs across five generations, in which all individuals were genotyped and phenotyped. One population consisted of Landrace pigs (3149 individuals) and the other population consisted of Yorkshire pigs (5053 individuals). We evaluated the decay in the prediction accuracy when the reference population was not updated and the prediction accuracy trends when individuals from only the immediate previous generation were used as the reference population. These were then compared to the prediction accuracy trends when individuals from all previous generations were used as the reference population. Additionally, prediction accuracy trends across generations were compared to the genomic relationships observed between the reference and test populations across generations.

## 2. Materials and Methods

A genetic model assuming additive SNP-effects was used for the genomic prediction of five traits (daily weight gain, weight at 90 days of age, average back fat, back fat depth, and meat percentage) in two populations of Korean pigs (Landrace (LL) and Yorkshire (YY), evaluated separately). Animals were genotyped on the Illumina PorcineSNP60 array (http://www.illumina.com). Quality control of the genotypes was performed with the program snpQC [13]. Individuals with call rates <0.9 were excluded from the data set, and only autosomal SNPs were considered for the analyses. SNPs with minor allele frequencies <0.01, with GC (GenCall) scores <0.6, or that failed in more than 5% of the samples were excluded from the genotype data. After quality control 47,225 SNPs remained for a total of 8202 individuals (3149 LL and 5053 YY). Missing SNP (<0.01%) were imputed with Eagle [14] using the software’s default parameters. Seven discrete generations (generation zero to generation six) were available in the populations, but generations four to six were combined into a single generation (4–6), due to the small number of individuals in generations five and six. Table 1 summarizes the number of individuals by breed per generation.

The phenotypes were pre-corrected for fixed effects (overall mean, sex, company, and parity) using a linear regression model, $y_{original}=Xb+e$, in which b were the coefficients for fixed effects and X their design matrix, and the pre-corrected phenotypes were defined as $y=y_{original}-X\hat{b}$. Then, as previously stated, we assumed an additive genomic model y=Ma+ε for the prediction of breeding values, in which y were the pre-corrected phenotypes, M the SNP genotypes, $a∼N\left(0,I\sigma_{a}^{2}\right)$ the additive random SNP effects, and $\varepsilon ∼N\left(0,I\sigma_{\varepsilon }^{2}\right)$ the random residuals. The breeding values were defined as g=Ma and the genomic relationship matrix (GRM) as $G=MM^{\prime }/\sum_{j=1}^{m}2p_{j}q_{j}$ [15], with $g∼N\left(0,G\sigma_{g}^{2}\right)$, $p_{j}^{\prime }s$ were the minor allele frequencies of the SNP genotypes ($q_{j}=1-p_{j}$), and $\sigma_{g}^{2}=\overset{m}{\underset{j=1}{\sum }}2p_{j}q_{j}\sigma_{a}^{2}$. Heritabilities ($h^{2}=\sigma_{g}^{2}/\left(\sigma_{g}^{2}+\sigma_{e}^{2}\right)$) for the entire dataset and for each generation group (Table 2) were estimated using the restricted maximum likelihood (REML) [16] and breeding values were estimated using GBLUP [15]. All analyses were coded and conducted with the R statistical programming language.

Along with the genomic evaluations across generations, we evaluated the genomic relationships between reference and test populations across these generations. For this evaluation, we calculated the mean values of the GRM between the reference and the test individuals ($mean(G_{ref,test})$), and we also calculated a genomic correlation between reference and test populations with a singular value decomposition (SVD) of the genotype matrix [17]. For this genomic correlation, we assumed $SVD\left(M\right)=UDV{}^{\prime }$ and $R=\sqrt{n_{test}/n_{ref}}V_{test}^{{}^{\prime }}V_{ref}D_{ref}$, with $SVD\left(R\right)=U_{R}TV_{R}{}^{\prime }$. R is a matrix that correlates the reference and the test individuals through their genotypes and accounts for their variance components and differences in population sizes. The prediction accuracy reaches its maximum ($\sqrt{h^{2}}$) when R and $D_{test}$ are similar matrices, and this similarity can be summarized as r=a+b, such that a and b are the coefficients of the regression $T∼D_{test}$. Because R is balanced for different population sizes, it is expected that −1<a<0 and 0<b<1. Thus, r=a+b is the correlation between reference and test populations using the SVDs of $M_{ref}$ and $M_{test}$ [17].

Prediction accuracy and the relationships between reference and test populations were obtained when the reference population was not updated (maintaining population zero as the reference for all subsequent generations), as well as when individuals from only the immediately previous generation were used as the reference population. A cumulative reference population with all individuals from previous generations was used to serve as the baseline (best case scenario). For each scenario, all animals available within these groupings where included in the analyses, there was no data sub-setting or resampling. Prediction accuracy trends across generations were then compared to the relationships between reference and test populations across generations.

## 3. Results

**Figure 1:**
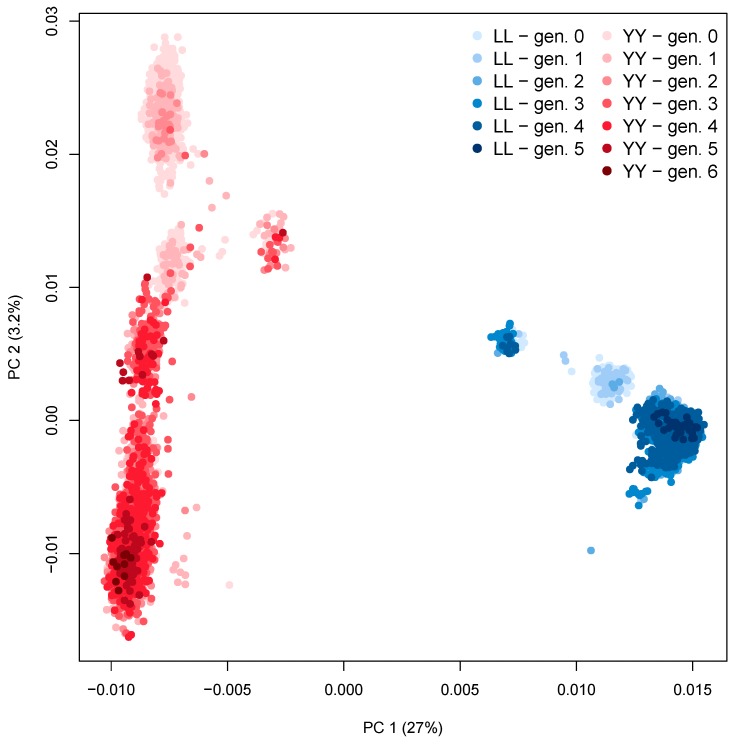
Principal components analysis of the genomic relationship matrix (GRM) by breed (Landrace (LL) and Yorkshire (YY)) and generations (zero to six).

We performed a brief analysis of the two pig populations (breeds LL and YY) and their generations, regarding the principal components of the GRM, and the phenotypic values. Figure 1 presents the first two principal components, that represent 27% and 3.2% of the total variance, respectively, by breed and generation. With the principal components analysis (PCA), we can observe a clear separation between the two breeds. Within breeds, the distinction between generations is less discrete, although a slight trend is discernable. The across generation variance for LL was 5.6% and for YY it was 9.2%, for the sum of the first two principal components of the GRM, when the PCA was performed on each breed independently.

**Figure 2:**
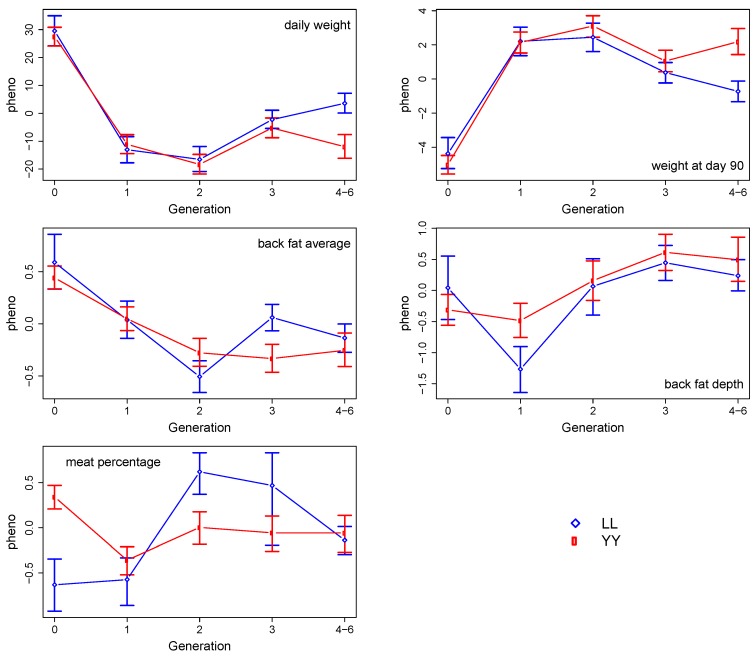
95% bootstrap confidence intervals from 1,000,000 resamples of the phenotypic values (corrected for fixed effects) by breed (Landrace (LL) and Yorkshire (YY)) and generations (zero to six).

Figure 2 presents the phenotypic values by breed and generations, for the five production traits (daily weight, weight at 90 days, average back fat, back fat depth and meat percentage). With the exception of meat percentage, the trends in the phenotypic values across the generations are very similar for both breeds.

Table 2 presents the heritabilities obtained with REML by breed and generations, for the five traits (daily weight, weight at 90 days, average back fat, back fat depth, and meat percentage). The standard deviation of heritability estimates ranged from 0.025 to 0.048.

**Figure 3:**
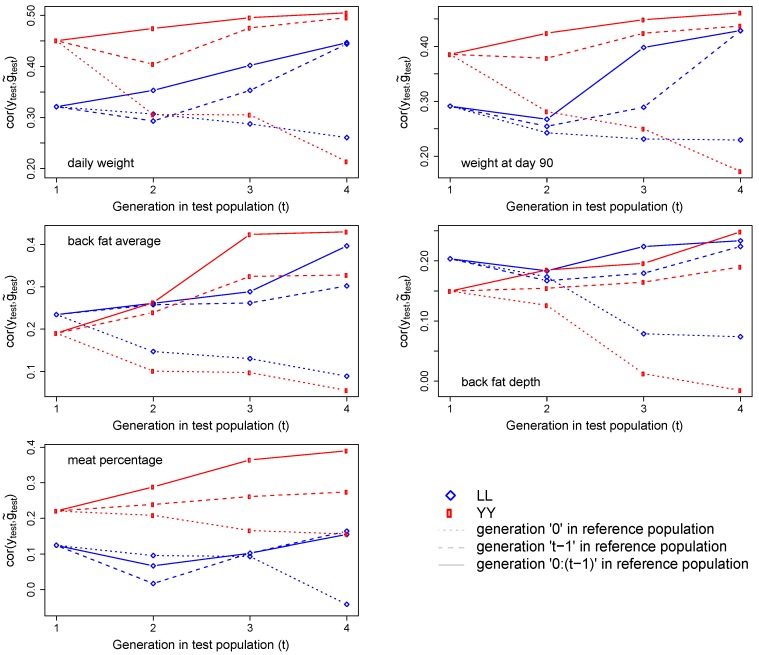
Prediction accuracies ($\hat{cor}\left(y_{test},{\tilde{g}}_{test}\right)$, the correlation between real phenotypes in the test population, $y_{test}$, and predicted breeding values, ${\tilde{g}}_{test}$ ) by breed (Landrace (LL) and Yorkshire (YY)) and generations (one to four, the last one combining generations four to six). Results are presented for the different generations considered as the reference population to perform genomic prediction, to predict a test generation (t).

Figure 3 presents the prediction accuracies obtained by breed and generations, for the five production traits (daily weight, weight at 90 days, average back fat, back fat depth, and meat percentage). We observed distinct levels of prediction accuracy when different generations were considered as the reference population. When generation zero was used as the reference to all subsequent generations, we observed a loss in prediction accuracy of the next generations for all traits, on both breeds. When the immediate previous generation or all the previous generations were used as reference populations, we observed an increase in prediction accuracy for all traits, in both breeds.

**Figure 4:**
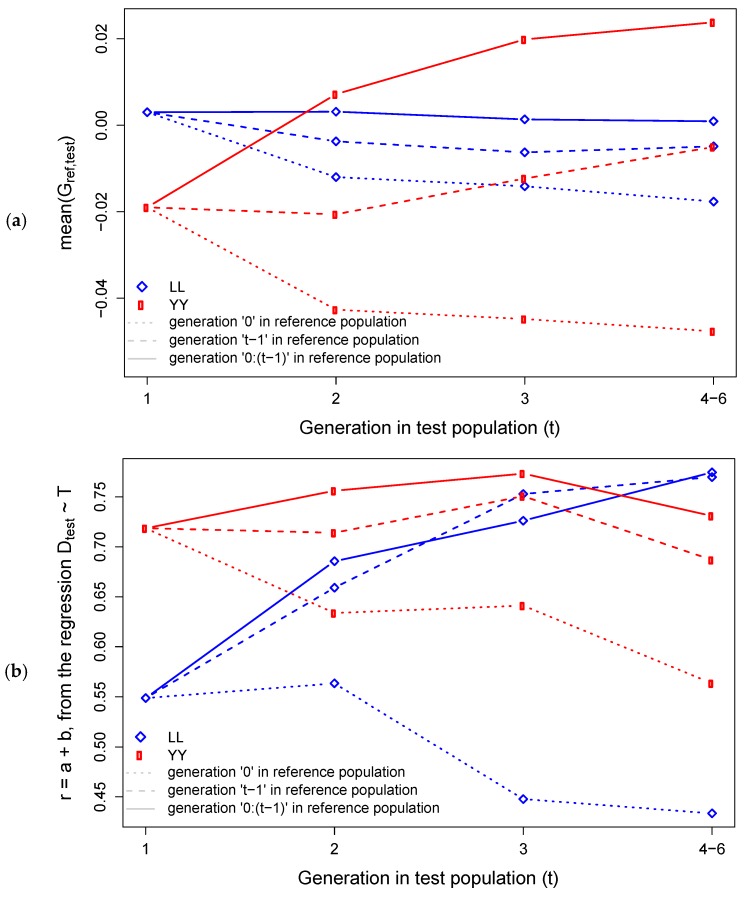
Genomic relationships between individuals in reference and test populations by breed (Landrace (LL) and Yorkshire (YY)) and generations (1 to 4–6). Panel (**a**) presents the average values in the off-diagonals of the GRM, and panel (**b**) presents the genomic correlation r=a+b, based on the singularvalue decomposition (SVD) of the genotype matrices.

Figure 4 presents the relationships between individuals in reference and test populations by breed and generations. We observed a distinction of the different generations considered as the reference population. When generation zero was fixed as the reference to all subsequent generations, we observed a decay in the relationships to further generations for all the traits, on both breeds. When the immediate previous generation was used as reference population, we observed that $mean(G_{ref,test})$ remained stable across generation for both breeds, while r=a+b increased for YY, and remained stable for LL. When all previous generation were used as reference population we observed that $mean(G_{ref,test})$ remained stable across generations for LL and increased for YY, while r=a+b increased for both LL and YY (with the exception of generation 4-6, on which there was a decrease of r=a+b for YY).

## 4. Discussion

Our study aimed to evaluate the accuracy of prediction across generations, and possibly relate trends in prediction accuracy to patterns in the relationships between individuals from reference and test populations. We did observe that the closer the individuals from reference and test populations are in terms of generations, both relationships and prediction accuracy increase.

While the close relationship between test and reference populations is highly relevant to prediction accuracy, it is also well known that sample size, and genetic and phenotypic variation are important contributors to genomic prediction. As expected, we did observe that when aggregating all past generations to the reference population, prediction accuracy indeed is higher than when only the immediately previous generation features in the reference population. This was intentional in this study as we primarily wanted to contrast the decay in accuracy across generations, and by using all previous aggregated generation data we could set the baseline of “best possible prediction” which could then be used to compare close versus further apart generations.

This same behavior in prediction accuracy was also observed in the relationships between individuals, and even clearer in our measure based on the SVD of the genotype matrices. The gain in prediction accuracy, from aggregating all past generations is, however, greater than the gain in the relationship between individuals. This is most likely a result of an additional gain in the prediction accuracy due to the greater size of the reference.

We did observe some fluctuations in the prediction accuracies, and this is most probably related to sample sizes. The number of individuals available for each generation in our data set was not homogeneous, and this may be a cause for the observed fluctuations. In addition, the scenario that incorporated all previous generations in the reference population represents the best-case scenario, serving as a comparative baseline for the prediction accuracies. An interesting result observed was that for LL, with the exception of back fat average, the prediction accuracy of generation 4–6 was very similar when either all previous generations were incorporated in the reference population, or only the immediately previous generation was used as reference. The same was observed for YY, for daily weight and weight at day 90. This indicates that potentially, if the population evolves mostly within itself, ancestral generations can be discarded from the reference over time, without compromising prediction accuracy. The sample sizes of the LL breed were quite small when compared to the YY breed, and the phenotypic values of LL did fluctuate more because of the small sample size, potentially resulting in less reliable prediction accuracies.

Cross-generational persistency of prediction accuracy should be further explored in animal breeding. One topic that should be addressed in future studies is how many generations back should a reference population incorporate, to predict the subsequent generation. We did observe that when all previous generations are aggregated to train the model, both the relationships between individuals and prediction accuracies start to reach a maximum limit. We did not however, have enough generations in our data set to explore this in detail. This is particularly interesting, in the sense that it can give a perspective of how much information is necessary to aggregate to reference populations, without adding too many individuals from ancient generations that do not incorporate any relevant information and increase the computer burden of performing the prediction models. Another topic that should be explored in these studies is the behavior of prediction accuracy in comparison to the intensity of selection, and the incorporation of new individuals into a herd.

While trends were observed in the prediction accuracy of the traits evaluated over generations (increasing accuracy of prediction when the reference population was updated, and decreasing accuracy of prediction when the reference populations was not updated), no clear or conclusive trends were observed on the estimated heritabilities. This is potentially a topic that would generate more discussion when combined with evaluations that explore intensity of selection, and the incorporation of new individuals into a herd.

Finally, comprehending how the accuracy of genomic prediction evolves over generations within a population may add extra and relevant information to improve the performance of genomic selection. Overall these results confirm the need to continuously update the reference with new genotypes and phenotypes, but they also show that it may not be necessary to keep all ancestral genotypes indefinitely in the reference population.

## 5. Conclusions

This study presented an empirical assessment of the prediction accuracy of genomic breeding values of production traits, across five generations in two Korean pig breeds. Overall, the results suggested that, although there is a clear need to continuously update the reference population, it may not be necessary to keep all ancestral genotypes. Finally, comprehending how the accuracy of genomic prediction evolves over generations within a population may add extra and relevant information to improve the performance of genomic selection.

## Figures and Tables

**Table 1:** Number of individuals by breed per generation (LL—Landrace, YY—Yorkshire).

Generation	Breed		
	LL	YY	Total
0	431	1475	1906
1	520	1172	1692
2	418	827	1245
3	916	987	1903
4	832	508	1340
5	32	74	106
6	0	10	10
all	3149	5053	8202

**Table 2:** Heritabilities of the five traits for different generations in reference populations, by breed (LL and YY).

Generation	Traits									
	Daily Weight		Weight at Day 90		Back Fat Average		Back Fat Depth		Meat Percentage	
	LL	YY	LL	YY	LL	YY	LL	YY	LL	YY
0	0.456	0.502	0.354	0.493	0.826	0.365	0.146	0.246	0.502	0.389
1	0.344	0.487	0.277	0.414	0.570	0.334	0.296	0.250	0.613	0.309
2	0.239	0.441	0.210	0.389	0.311	0.364	0.305	0.246	0.515	0.374
3	0.361	0.435	0.344	0.412	0.316	0.486	0.254	0.164	0.310	0.456
4–6	0.346	0.431	0.298	0.403	0.305	0.424	0.295	0.237	0.289	0.397
0–1	0.373	0.485	0.291	0.456	0.634	0.334	0.193	0.196	0.476	0.336
0–2	0.323	0.490	0.251	0.450	0.579	0.347	0.199	0.206	0.426	0.331
0–3	0.326	0.494	0.271	0.458	0.481	0.392	0.192	0.200	0.311	0.345
0–6	0.332	0.492	0.282	0.453	0.466	0.385	0.193	0.201	0.308	0.337
